# Physical therapy for the treatment of frozen shoulder: A protocol for systematic review of randomized controlled trial: Retraction

**DOI:** 10.1097/MD.0000000000042727

**Published:** 2025-06-06

The article, “Physical therapy for the treatment of frozen shoulder: A protocol for systematic review of randomized controlled trial”,^[1]^ which appears in Volume 98, Issue 32 of *Medicine*, is being retracted over concerns with the methodology. After publication, concerns were raised over references to the “Begger test,” which does not exist in the literature and is indicative of possible fraud. Upon further investigation, authorship irregularities were also discovered in our submission system. As a result, the Publisher no longer has confidence in the integrity of the content or the authorship.
